# Midterm functional recovery of Total knee arthroplasty patients compared between the ATTUNE knee system and the press fit condylar (PFC) SIGMA knee system

**DOI:** 10.1186/s12891-021-04464-6

**Published:** 2021-07-13

**Authors:** Ekasame Vanitcharoenkul, Aasis Unnanuntana

**Affiliations:** Department of Orthopedic Surgery, Faculty of Medicine Siriraj Hospital, Mahidol University, 2 Wanglang Road, Bangkoknoi, Bangkok, 10700 Thailand

**Keywords:** Functional recovery, Total knee arthroplasty, TKA, ATTUNE, Press fit condylar, PFC

## Abstract

**Background:**

The ATTUNE Knee System is a new prosthetic design that has theoretical advantages over the Press Fit Condylar (PFC) Sigma Knee System specific to improved knee kinematics and patellofemoral joint tracking. This study aimed to evaluate functional outcomes compared between the ATTUNE and PFC Sigma designs at a minimum follow-up of 5 years.

**Methods:**

We retrospectively reviewed data from total knee arthroplasty (TKA) patients who received either the ATTUNE or PFC Sigma system during November 2013 to February 2015 at Siriraj Hospital (Bangkok, Thailand). Functional outcomes were evaluated using Timed Up and Go (TUG) test, 2-min walk test (2MWT), modified knee score, numerical rating scale-pain, range of motion (ROM), and rate of anterior knee pain and crepitation at preoperation, 3-months, 1-year, and 5-years postoperatively.

**Results:**

Of 113 patients, 59 and 54 received the PFC Sigma and ATTUNE systems, respectively. At a minimum of 5-years follow-up, all functional outcomes improved significantly from the preoperative period although TUG test and 2MWT declined significantly from 1- to 5-years postoperatively only in the PFC Sigma group. The mean ROM at 5-years postoperatively was significantly higher in ATTUNE than in PFC Sigma; however, the difference was small (116° vs. 110°, respectively; *p* = 0.041). There were no significant differences in any of the other outcome measurements, including anterior knee pain, clunking, and crepitation, between groups at any study time point.

**Conclusions:**

​Our results revealed no major differences in functional outcomes between the PFC Sigma and ATTUNE TKA designs at an intermediate-term follow-up of at least 5 years. Longer-term follow-up study is needed to evaluate the benefits of the ATTUNE design relative to polyethylene wear and the rate of aseptic loosening.

**Supplementary Information:**

The online version contains supplementary material available at 10.1186/s12891-021-04464-6.

## Background

Total knee arthroplasty (TKA) is a highly successful orthopedic surgical procedure that delivers pain relief, functional restoration, and enhanced mobility [1–3]. Having acknowledged the commonly realized benefits of TKA, several studies have reported unsatisfactory outcomes after TKA, including unsatisfactory postoperative functional scores [4], patellofemoral complication [5], and residual pain [6]. Previous studies reported rates of dissatisfaction that ranged from 10 to 20% [4–6].

To improve patient satisfaction, several different knee prosthesis designs have been developed. The Press Fit Condylar (PFC) Sigma Knee System (DePuy Synthes, Warsaw, IN) (hereafter PFC Sigma), which is one of the most commonly used TKA designs, was initially introduced with a dome-shaped patella and a single radius trochlear groove design in 1996 [7, 8]. This prosthesis is available with a range of options, including fixed-bearing (FB), rotating-platform (RP), cruciate-retaining (CR), and posterior-stabilized (PS) components. This TKA system has shown excellent long-term survivorship [9, 10].

The ATTUNE Knee System (DePuy Synthes) (hereafter ATTUNE) is a newer-generation TKA that was introduced in 2013 with modifications to the following femoral and tibial components: gradually reducing radius from distal to posterior condyle of the femoral component, reduced femoral component profile, improved locking mechanism of the tibial base, improved anatomic trochlear groove, and medialized patellar shape. It is thought that these design modifications, which more closely replicate the anatomy of the human knee, can reduce pain and crepitation leading to better functional outcome and improve patient satisfaction. However, the results of comparisons between the ATTUNE knee system and other TKA prostheses remain limited. In one study, patient-reported outcomes of the ATTUNE TKA showed superior results over those of the PFC Sigma at 6 months after surgery; however, the number of recruited patients was small, and the follow-up period was short [11]. Another study, which was conducted by the pioneer designer of the ATTUNE TKA, concluded that the ATTUNE TKA could reduce the incidence of anterior knee pain and crepitation compared with the PFC Sigma prosthesis with no differences in Knee Society Score (KSS) or patient satisfaction [7]. In addition to the aforementioned paucity of comparative ATTUNE TKA data, no studies have compared performance-based outcomes, which are believed to truly reflect the actual function, balance, and walking ability of patients, between the ATTUNE TKA design and the PFC Sigma design.

Accordingly, the objective of this study was to compare functional outcomes, specifically performance-based measures, between the ATTUNE and PFC Sigma TKA knee systems at a minimum follow-up of 5 years. We hypothesized that patients who received the ATTUNE TKA system would have better postoperative functional performance than those who received the PFC Sigma system.

## Methods

The protocol for this study was reviewed and approved by the Siriraj Institutional Review Board (SIRB) of the Faculty of Medicine Siriraj Hospital, Mahidol University, Bangkok, Thailand (COA no. 726/2016). A retrospective review of data from a cohort group of patients who were diagnosed with end-staged osteoarthritis (OA) of the knee and who underwent primary unilateral TKA at our center during November 2013 to February 2015 was performed. Patients aged 40–100 years who received TKA with either the ATTUNE or PFC Sigma TKA system were eligible for inclusion. Patients did not consecutively receive either the ATTUNE or PFC Sigma TKA system. Selection of the prostheses in each patient was based on both the surgeon’s discretion and the patient’s ability to cover the extra cost of the ATTUNE system. Patients who had symptomatic knee OA on the contralateral side; history of high tibial osteotomy or who underwent a complex surgical procedure that required a metal augment or stem during the operation; and/or, who had complications or a severe medical condition that affected the standard rehabilitation protocol were excluded.

### Operative procedure and postoperative care

All surgeries were performed by a single surgeon (AU) using a medial parapatellar approach. A tourniquet was applied in all cases. Standard surgical techniques were performed in all cases, including midline skin incision, subluxation of the patella, intramedullary distal femoral resection, and extramedullary proximal tibial resection. Appropriate size and rotation of the femoral component was determined using anterior referencing and the gap balancing technique, respectively. The patella was selectively resurfaced, as needed, according to the intraoperative findings. If cartilage on the patella was generally preserved with adequate patellofemoral congruency, and there was no history of crystalline or inflammatory synovitis, the patella was not resurfaced [12]. All components were cemented posterior-stabilized and fixed-bearing design. Antibiotic was given for approximately 24 h after surgery. Intravenous fluid and Foley catheter were typically removed on the second postoperative day. Following TKA, all patients received the same postoperative rehabilitation protocol, including early mobilization and full weight-bearing with a walker as soon as possible. Postoperative pain was controlled with a combination of oral analgesics, including acetaminophen, non-steroidal anti-inflammatory drugs (NSAIDs), muscle relaxant, and oral opioids, as needed. Intravenous morphine was administered when the patient’s pain score was ≥5. The goal was to maintain the patient’s pain score at no higher than 2–3 out of 10 as measured by numerical rating scale-pain (NRS-pain). Deep vein thrombosis prophylaxis was performed using mechanical prophylaxis, and medical prophylaxis if indicated. Patients were discharged home and instructed to perform simple knee exercises using a walker a few times a day for approximately 6 weeks.

### Assessment of outcomes

Preoperative demographic data and clinical information were collected, including age, sex, body mass index (BMI), side of the operation, Charlson Comorbidity Index (CCI), use of walking assisting device, preoperative alignment, range of motion (ROM), and modified knee score. Alignment of the knee was determined using hip knee axis (HKA), and alignment was classified as varus alignment if the HKA was <178^o^, and valgus alignment if the HKA was >182^o^ [13]. Our primary outcome of interest was the performance-based tests: Timed Up and Go (TUG) test and 2-min walk test (2MWT). The secondary outcomes included NRS-pain, ROM, modified knee score, postoperative complications, and the percentage of patients who reported crepitation, patellar clunk, or anterior knee pain. All outcome measures were collected during the preoperative period as baseline data, and at 3-months, 1-year, and 5-years postoperatively.

### Timed up and go (TUG) test

The TUG test is a reliable and validated outcome that has been used to evaluate the physical performance of patients with TKA [14]. Participants were asked to stand up from a seated position on a standard chair, walk 3 m with or without an assistive device, turn around, walk back to the chair, and then sit back down. They were allowed to use their arms when rising from and returning to a seated position. The time used to perform this task was measured in seconds [14, 15]. Patients were asked to perform this test twice with a 5-min rest interval between tests, and the average time was used for analysis.

### Two-minute walk test (2MWT)

Patients were instructed to walk for two minutes at their normal pace up and down a designated corridor, turning around at each end of the corridor without stopping [16]. They were permitted to use a walking aid if they wanted to do so. Patients were asked to perform this test once. The results were recorded as total distance walked in meters. Similar to the TUG test, the 2MWT has been validated to evaluate function after TKA [17].

### Modified knee score

This scoring system was modified from the original Knee Society Clinical Rating System that was published by Insall in 1993 [18]. The modified knee scoring system is based on 3 aspects: pain, ROM, and stability. The summation of the individual scores from each aspect is then reduced by the total of the scores given for extension lag, flexion contracture, malalignment, and pain at rest. The resulting score is the patient’s overall modified knee score. The highest possible score is 100 points, with higher scores indicating better knee condition, and lower scores indicating worsened knee condition.

### Numerical rating scale-pain (NRS-pain)

The level of postoperative knee pain experienced by patients while performing daily activities was assessed using the NRS-pain score. NRS-pain is a subjective measurement of pain on an eleven-point numerical scale that ranges from 0 (no pain) to 10 (the worst imaginable pain). NRS-pain is efficient for use in clinical practice, and showed good test-retest reliability in different populations [19].

### Crepitation, patellar clunk, and anterior knee pain

Patellofemoral crepitus or crepitation is defined as painless hearable noises and/or palpable vibrations from the knee of a TKA patient. Patellar clunk was first described by Hozack, et al. [20], and is defined as a painful catching, grinding, or jumping of the patella when the knee moves from a flexed to an extended position after total knee replacement [21]. Anterior knee pain is a subjective symptom of pain around the anterior part of the knee [22]. Patients were determined to have anterior knee pain if they reported pain in front of their knee. The occurrence of any noise and/or anterior knee pain was determined in a dichotomized fashion (Yes/No) at each follow-up time point.

### Statistical analysis

Based on the minimal clinically important difference (MCID) reported in a previous study [17], a power analysis conducted a priori determined that a minimum of 46 and 34 subjects per group was required to establish a minimum effect size of the MCID values for TUG test and 2MWT, respectively. Therefore, the minimum number of subjects required for this study was set at 46 patients per group with a 2-sided alpha level of 0.05 and 90% power for both performance-based tests. Each variable and outcome measure was assessed for normality using Shapiro-Wilk test. Data are presented as mean and standard deviation for continuous variables, and as frequency and percentage for categorical variables. Student’s unpaired *t*-test was used to compare quantitative variables and differences between the 2 prosthesis systems at each follow-up time point. Condition of equal variance was verified by Levene’s test. Chi-square test was used to compare categorical variables between groups. The rate of patients who had anterior knee pain and crepitation was analyzed using Fisher’s exact test. Analysis of covariance (ANCOVA) and Levene’s test were used to compare the results and verify the variance in each outcome measurement from baseline to 3 months, 3 months to 1 year, and 1 year to 5 years within one study group. Normally distributed residual outcomes in ANCOVA were assessed using Shapiro-Wilk test. Statistical analyses were performed using SPSS Statistics for Windows, version 18.0 software (SPSS, Inc., Chicago, IL, USA). Statistical significance was defined as a *p*-value less than 0.05.

## Results

From a total of 155 patients, 42 were excluded due to the following conditions: symptomatic contralateral knee OA (*n* = 25), received TKA using other prosthesis designs (*n* = 14), active spinal disease (n = 1), underwent complex primary TKA (n = 1), and had periprosthetic fracture at 2 months after the index surgery (n = 1). The remaining 113 patients were enrolled in this study. Of those, 59 and 54 patients underwent TKA using the PFC Sigma and ATTUNE knee systems, respectively. Nine patients underwent patellar resurfacing; 6 patients (10.2%) and 3 patients (5.6%) were in the PFC Sigma and the ATTUNE groups, respectively (*p* = 0.366). The mean follow-up duration was 75.3 months (range: 60.0 to 92.0). From a total of 59 patients who received the PFC Sigma system, 57, 56, and 55 patients completed 3 months, 1 year, and 5 years of follow-up post-TKA, respectively. In the ATTUNE group, 54, 51, and 53 patients completed 3 months, 1 year, and 5 years of follow-up post-TKA, respectively.

Preoperative patient demographic and clinical characteristics are shown in Table 1. The average age of patients before surgery was 71.1 years, and most of them (89.4%) were female. The majority of patients had CCI in the range of 0–3 in the PFC Sigma group (59.3%), and higher than 3 in the ATTUNE group (55.6%). There were no significant differences in sex or CCI distribution between the two groups. Most patients had varus knee alignment before TKA (81.4%). The mean preoperative HKA among all patients was 169.9 degrees. There were no significant preoperative differences between groups relative to gait aid use, knee alignment, ROM, or modified knee score.

**Table 1:** Preoperative patient demographic and clinical characteristics

Characteristics	PFC Sigma (***n*** = 59)	ATTUNE (***n*** = 54)	***p***-value
Age (years), mean ± SD	70.0 ± 8.2	73.4 ± 7.2	***0.023***
(min, max)	(54.0, 86.0)	(54.0, 87.0)	
Female sex, n (%)	51 (86.4%)	50 (92.6%)	0.367
Body mass index (kg/m^2^), mean ± SD	26.5 ± 4.9	26.2 ± 3.7	0.679
(min, max)	(16.9, 39.6)	(19.0, 38.9)	
Right side, n (%)	30 (50.8%)	23 (42.6%)	0.452
Charlson Comorbidity Index, n (%)			0.114
0–3	35 (59.3%)	24 (44.4%)	
> 3	24 (40.7%)	30 (55.6%)	
Preoperative use of gait aid, n (%)			0.098
None	33 (56.0%)	20 (37.0%)	
Cane	13 (22.0%)	17 (31.5%)	
Walker	12 (20.3%)	17 (31.5%)	
Wheel chair	1 (1.7%)	0 (0.0%)	
Preoperative Hip knee axis (degrees), mean ± SD	170.0 ± 10.9	169.8 ± 8.1	0.912
(min, max)	(149.0, 199.0)	(153.0, 197.0)	
Varus alignment, n (%)	47 (79.7%)	45 (83.3%)	
Valgus alignment, n (%)	7 (11.9%)	2 (3.7%)	
Preoperative range of motion (degrees), mean ± SD	102.5 ± 18.2	100.5 ± 16.3	0.538
(min, max)	(30.0, 140.0)	(50.0, 130.0)	
Preoperative modified knee score, mean ± SD	44.9 ± 11.4	44.9 ± 10.2	0.978
(min, max)	(7.0, 67.0)	(25.0, 70.0)	

Student’s unpaired *t*-test was used to compare quantitative variables between two groups, whereas chi-square test was used to compare categorical variables

A *p*-value< 0.05 indicates statistical significance

Varus alignment is defined as tibiofemoral angle <178^o^

Valgus alignment is defined as tibiofemoral angle >182^o^

**Abbreviation:** PFC, Press Fit Condylar; SD, standard deviation; min, minimum; max, maximum

**Fig. 1:**
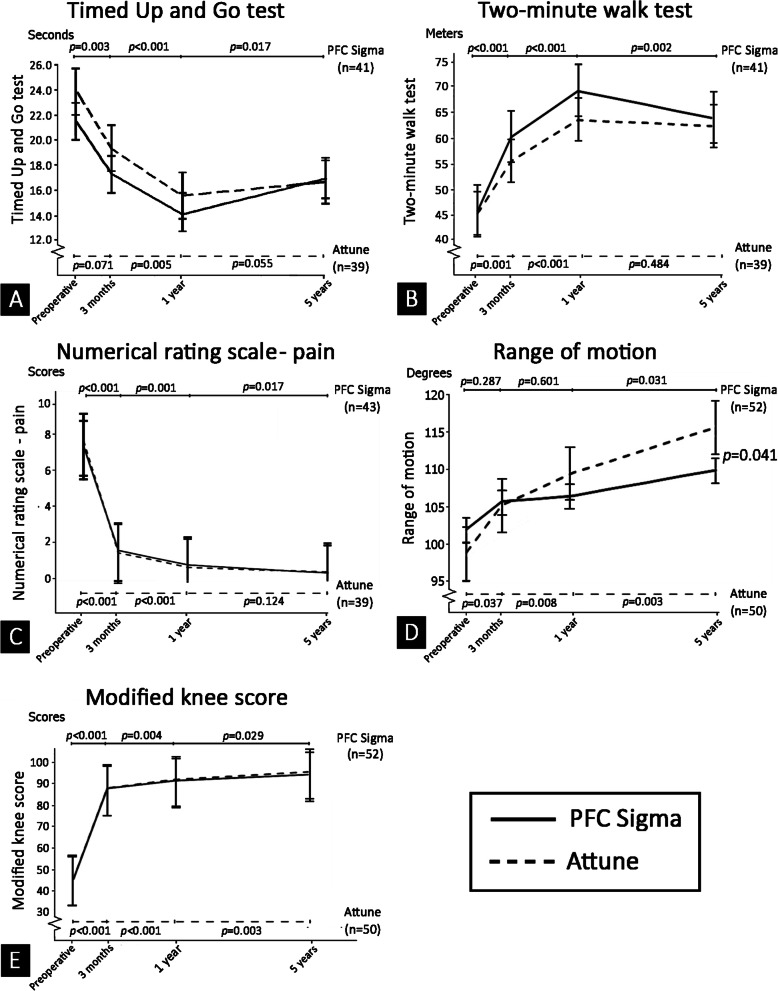
Mean values of each outcome measurement, and within group *p*-values to compare data at baseline, 3 months, 1 year, and 5 years postoperatively. (A) Timed Up and Go test; (B) Two-minute walk test; (C) Numerical rating scale - pain; (D) Range of motion; and, (E) Modified knee score (The numbers (n) in each graph indicate the number of patients with complete follow-up data at all time points. The data were analyzed by using analysis of covariance (ANCOVA). A *p*-value represents a comparison of each outcome between the current time point and the previous time point)

The mean preoperative performance-based test results in the PFC Sigma and ATTUNE groups were 21.5 and 23.9 s for the TUG test, and 45.8 and 45.3 m for the 2MWT, respectively. At 1-year post-TKA, the time needed to complete the TUG test was reduced to 14.3 and 15.6 s in the PFC Sigma and ATTUNE groups, respectively. Similarly, the distance walked within 2 min increased to 69.4 and 63.8 m in the PFC Sigma and ATTUNE groups, respectively, at the 1-year follow-up time point (Table 2). When using ANCOVA to analyze data from only patients who had complete results at all time points (baseline, 3-months, 1-year, and 5-years post-TKA), the performance-based measures declined significantly from 1- to 5-years post-TKA only in the PFC Sigma group (*p* = 0.017 and *p* = 0.002 for TUG test and 2MWT, respectively) (Fig. 1). However, there were no significant differences in these two performance-based measures between the PFC Sigma and ATTUNE groups at any study time point (Table 2). When evaluating only patients who used a gait aid preoperatively, we found no differences in TUG test or 2MWT between these 2 prosthesis designs (Supplementary Table 1 and Supplementary Fig. 1)*.* Additional subgroup analysis to evaluate the effect of preoperative CCI on changes in TUG test and 2MWT results showed no differences in these two performance-based outcomes between the PFC Sigma and ATTUNE systems in the subgroup of patients who had preoperative CCI 0–3, and in those who had preoperative CCI > 3 (Supplementary Table 2).

**Table 2:** Outcome measurements compared between the PFC Sigma and ATTUNE systems at baseline, and at the 3-month, 1-year, and 5-year postoperative follow-ups

Outcome measurement	PFC Sigma (***N*** = 59)* Mean ± SD	ATTUNE (***N*** = 54)* Mean ± SD	***p***-value
**Timed up and go test**			
Baseline (n = 59/54)**	21.5 ± 10.9	23.9 ± 14.8	0.425
3 months (*n* = 55/53)**	17.3 ± 7.2	19.4 ± 9.2	0.276
1 year (n = 55/47)**	14.3 ± 5.6	15.6 ± 5.6	0.322
5 years (*n* = 41/39)**	16.9 ± 9.9	16.8 ± 5.6	0.949
**Two-minute walk test**			
Baseline (n = 59/54)**	45.8 ± 21.5	45.3 ± 18.5	0.919
3 months (n = 55/53)**	60.4 ± 20.7	55.6 ± 18.2	0.293
1 year (n = 55/47)**	69.4 ± 19.0	63.8 ± 15.6	0.164
5 years (n = 41/39)**	64.1 ± 21.5	62.5 ± 16.2	0.709
**Numerical rating scale-pain**			
Baseline (n = 59/54)**	7.4 ± 1.9	7.7 ± 1.6	0.447
3 months (*n* = 56/54)**	1.6 ± 1.3	1.5 ± 0.9	0.560
1 year (n = 56/51)**	0.8 ± 1.2	0.6 ± 0.7	0.561
5 years (*n* = 44/41)**	0.3 ± 0.6	0.4 ± 0.8	0.734
**Range of motion (degrees)**			
Baseline (n = 59/54)**	102.0 ± 21.6	98.9 ± 13.6	0.522
3 months (n = 55/53)**	105.7 ± 11.2	105.3 ± 7.6	0.855
1 year (n = 55/50)**	106.5 ± 10.3	109.6 ± 7.9	0.203
5 years (n = 55/50)**	110.0 ± 10.8	115.8 ± 9.9	***0.041***
**Modified knee score**			
Baseline (n = 59/54)**	44.9 ± 11.3	45.2 ± 10.5	0.884
3 months (*n* = 57/54)**	87.2 ± 6.5	87.5 ± 4.1	0.761
1 year (n = 56/51)**	90.9 ± 7.9	91.4 ± 6.1	0.696
5 years (n = 55/53)**	93.8 ± 6.4	95.0 ± 4.2	0.276

*N indicates the total number of patients before surgery in each group

**(n/n) indicates the number of patients at each follow-up time point in the PFC Sigma and ATTUNE groups, respectively

Student’s unpaired *t*-test was used to compare differences in each outcome between the PFC Sigma and ATTUNE systems at each follow-up time point

A *p*-value< 0.05 indicates statistical significance

**Abbreviation:** PFC, Press Fit Condylar; SD, standard deviation

Postoperative NRS-pain score and modified knee score were both significantly improved during the first year after surgery. Afterwards, the mean NRS-pain score in the PFC Sigma group significantly improved from 0.8 point at 1-year post-TKA to 0.3 point at 5-years post-TKA (*p* = 0.017); however, there was no significant change in the NRS-pain score in the ATTUNE group (*p* = 0.124) (Fig. 1). There was also no significant difference in the NRS-pain score or the modified knee score between these two prosthetic designs at any study time point. Although the 1-year postoperative ROM increased from the preoperative period, the difference was statistically significant only in the ATTUNE TKA group. Thereafter, ROM continued to improve throughout the 5-year postoperative period in both study groups (Fig. 1). The mean postoperative ROM at 5-years post-TKA was significantly higher in the ATTUNE group than in the PFC Sigma group (115.8 and 110.0 degrees, respectively; *p* = 0.041) (Table 2).

Across all time points, postoperative anterior knee pain was observed in 1 patient, and that patient received a PFC Sigma. Crepitation was found in 6 patients in both groups. There was no significant difference between groups relative to anterior knee pain (*p* = 1.000) or crepitation (*p* = 0.423). No patients in this study developed patellar clunk syndrome at any time point. There were 6 patients who developed stiffness after TKA that required manipulation under anesthesia at 3 months after surgery. One patient in the PFC Sigma group had wound dehiscence that required debridement and resuturing. No patients in this study developed aseptic loosening or superficial/deep infection after TKA (Table 3).

**Table 3:** Cumulative results of anterior knee pain, crepitation, patellar clunk, and postoperative complications from all follow-up time points

Outcome measurement	PFC Sigma (***n*** = 59)	ATTUNE (n = 54)	***p***-value
Anterior knee pain, n (%)	1 (1.7%)	0 (0.0%)	1.000
Asymptomatic crepitation, n (%)	2 (3.4%)	4 (7.4%)	0.423
Patellar clunk, n (%)	0 (0.0%)	0 (0.0%)	N/A
Complication, n (%)	3 (5.1%)	4 (7.4%)	0.708
- Stiff knee	2 (3.4%)	4 (7.4%)	
- Wound dehiscence	1 (1.7%)	0 (0.0%)	

Fisher’s exact test was used to compare differences between the two prosthesis designs

A *p*-value< 0.05 indicates statistical significance

**Abbreviation:** PFC, Press Fit Condylar; N/A, not applicable

## Discussion

The ATTUNE TKA system, which represents an advancement in knee replacement technology, was expected to deliver better performance than the PFC Sigma TKA system. However, the results of our study revealed no significant differences between the ATTUNE and PFC Sigma knee systems relative to pain, modified knee score, or performance-based measures during a minimum follow-up of 5 years. White, et al. [23] conducted a combined retrospective and prospective cohort matched-pair study that found significantly less residual pain (using yes or no questions) in the ATTUNE group than in the PFC Sigma group at the 5-year follow-up (19.5% vs. 36.3%, respectively; *p* = 0*.*02). However, when KSS and the Western Ontario and McMaster University Osteoarthritis Index (WOMAC) were compared between the two knee systems, no significant difference between groups was observed for either measure. In addition – in those who reported residual pain, the pain symptom was only mild and occasional. Therefore, this residual pain might not be clinically important. Similar to our results, they reported no significant differences in functional outcomes, including the rate of anterior knee pain, crepitation, or modified knee score, between the two groups.

Previous studies showed that the post-TKA ROM plateaued at approximately 6 to 12 months postoperatively [24, 25]. Conversely, our study showed that the ROM continued to increase from 1-year to 5-years postoperatively in both evaluated knee systems. This could be due to design differences between the 2 prosthetic systems or the significant difference in mean age between the 2 groups. As people get older, there is a tendency for soft tissue to become more relaxed, which can lead to increased ROM [26]. Additionally, the mean ROM in the ATTUNE group was significantly higher than that of the PFC Sigma group (115.8 vs. 110 degrees, respectively; *p* = 0.041) at 5-years post-TKA; however, the 5.8-degree gain in the ATTUNE TKA group may not be clinically important. It is also important to point out that there was a significant decline in performance-based measures from 1- to 5-years post-TKA only in the PFC Sigma group. The reason for better ROM at 5 years and the maintenance of performance-based measures in the ATTUNE group is still unclear. It is possible that some design modifications that were engineered into the ATTUNE system might have improved or helped to maintain knee kinematics, which may have effectuated late improvement in ROM and maintenance of function compared to that of the PFC Sigma. Since ROM is multifactorial, further investigation to explore this finding is needed.

Although most previous studies found no major differences between the ATTUNE and PFC Sigma systems relative to functional outcomes during a short-term follow-up [27–30] (Table 4), some studies reported that the ATTUNE system had less patients who experienced crepitation and pain [7, 23, 31, 32] due to improvement in some design features, including modifications to the trochlear groove and patellar shape to prevent patellofemoral maltracking, patellar tilt, and patellofemoral overstuffing [7]. Martin, et al. [32] found a lower rate of crepitation in the ATTUNE group than in the PFC Sigma group at the 2-year follow-up (0.83% vs. 9.4%, respectively). Ranawat, et al. [7] reported that ATTUNE patients had less incidence of anterior knee pain and crepitation than PFC Sigma patients; however, a longer-term follow-up study at 5-years postoperatively of the same population found no significant difference in the incidence of anterior knee pain between groups (11.7% vs. 22.1% for the ATTUNE and PFC Sigma groups, respectively; *p* = 0.09) [23]. The authors suggested explanation for this finding was that some patients in the earlier cohort were diseased and lost to follow-up. Thus, the 5-year study might have been underpowered to detect a difference in the occurrence of anterior knee pain. Song, et al. [33] reported that the ATTUNE group had a significantly higher knee score domain in the KSS (93.1 vs. 88.8 points for ATTUNE and PFC Sigma respectively, *p <* 0.001), and that ATTUNE yielded approximately 2.4 degrees more ROM than the PFC Sigma at the 2-year follow-up. This difference, however, was still within the 7.2 points of the MCID of the KSS [34]. It is also important to point out that none of those previous studies evaluated functional outcome using performance-based testing like the TUG test and 2MWT. Since patient-reported outcome measures (PROMs) and performance-based testing are interchangeable and equally important [35], a comprehensive evaluation of knee function should include a combination of PROM and performance-based tests to better assess functional recovery in patients after TKA.

**Table 4:** Literature review for previous studies that compared the outcome results between the PFC Sigma and ATTUNE knee systems

Paper	Year of publication	Type of study	Duration	Sample size	Outcomes	Findings
Indelli, et al. [31]	2016	Prospective, matched-pair	2 years	200	KSS, ROM, Oxford knee score, satisfaction rate	ATTUNE had a significantly lower incidence of anterior knee pain (2% vs. 9%, *p =* 0.006), but significantly higher ROM (123 degrees vs. 115 degrees, *p =* 0.0009)
Ranawat, et al. [7]	2017	Prospective, matched-pair	2 years	200	KSS, anterior knee pain, crepitation, satisfaction rate	ATTUNE had a significantly lower incidence of anterior knee pain (12.5% vs. 25.8%, *p =* 0.02) and crepitation (17.7% vs. 30.9%, *p =* 0.02)
Martin, et al. [32]	2017	Retrospective	2 years	1893	KSS, ROM, crepitation	ATTUNE had a significantly lower incidence of crepitation (0.8% vs. 9.4%, *p <* 0.001)
Song, et al. [33]	2018	Prospective	2 years	600	KSS, ROM, risk of patella injury	ATTUNE had a significantly higher knee score domain in the KSS (93.1 vs. 88.8 points, *p <* 0.001) and ROM (131.4 vs. 129.0 degrees, *p =* 0.008)
Chua, et al. [28]	2019	Prospective	2 years	130	KSS, ROM, Oxford knee score, Short Form-36, satisfaction rate	No significant differences in any outcome measurements
Molloy, et al. [29]	2019	Prospective	2 years	2116	Patient-reported outcome measures, ROM, reoperative rate	No significant differences in any outcome measurements
Hauer, et al. [27]	2020	Randomized control trial	2 years	158	KSS, ROM, WOMAC, VAS	No significant differences in any outcome measurements
White, et al. [23]	2020	Combined prospective & retrospective, matched-pair	5 years	154	KSS, WOMAC, anterior knee pain, crepitation, residual pain, satisfaction rate	ATTUNE had a significantly lower incidence of residual pain (19.5% vs. 36.3%, *p =* 0.02*)*
Maniar, et al. [30]	2020	Prospective, matched-pair	2 years	144	New KSS, ROM, WOMAC, anterior knee pain, crepitation	No significant differences in any outcome measurements
The present study		Retrospective	5 year	113	Modified knee score, ROM, NRS-pain, TUG, 2MWT, anterior knee pain, crepitation, patellar clunk	ATTUNE had a significantly higher ROM at the 5-year postoperative follow-up (115.8 vs. 110.0 degrees, *p =* 0.041)

**Abbreviations:** PFC, Press Fit Condylar; KSS, Knee Society Score; ROM, range of motion; WOMAC, Western Ontario and McMaster Universities Osteoarthritis Index; VAS, visual analog scale for pain; TUG, timed up and go test; 2MWT, two-minute walk test; NRS-pain, numerical rating scale-pain

The strength of this study is that it is the first to compare the performance-based outcomes of two commonly used TKA designs at a minimum follow-up of 5 years. Our study also has some mentionable limitations. First, as with all retrospective studies, our study was subject to inherent biases in patient selection. Second, the study period was considered intermediate (range: 60 to 92 months), which may not be long enough to prove the longevity of these two prostheses. Further follow-up is needed to compare the survivorship of these two knee systems in this patient population. Third, our study used modified knee score, ROM, NRS-pain and two performance-based tests as outcome measurements. However, other knee functional scores and performance-based tests are available, and we are not able to recommend which of those metrics are best for evaluating function after TKA. The scores and tests that we used in this study may not be sensitive enough to detect statistically significant differences in knee function between the ATTUNE and PFC Sigma TKA designs. It is possible that patients with one prosthetic design could have had better postoperative function if they were asked to perform above the limits of the functional tests that were used in this study. Fourth, since all data were collected retrospectively from patient medical charts, some essential information, such as preoperative activity level and the results at some follow-up time points were missing. In addition, we didn’t record whether each patient had performed the performance-based tests with or without a gait aid. Instead, we assumed that those who had been using a walking assisting device preoperatively would have used a gait aid during the TUG test and 2MWT. Lastly, we calculated our sample size based on the MCID of the TUG test at 1-year post-TKA. Post hoc analysis to evaluate power for other secondary outcome measures showed 87, 99, 59.1, and 50.5% power for 2MWT, NRS-pain, range of motion, and modified knee score, respectively. Therefore, our study is underpowered to detect differences in range of motion and modified knee score at 1-year post-TKA between the two prosthesis designs.

## Conclusions

The results of this study revealed no significant differences in performance-based measures or other functional outcomes, including anterior knee pain, clunking, or crepitation, between the ATTUNE and PFC Sigma knee systems at a minimum follow-up of 5 years after TKA even though the ATTUNE design is thought to possess theoretical advantages over the PFC Sigma design. It remains unknown whether the new polytheylene in the ATTUNE TKA will reduce wear and the rate of aseptic loosening; therefore, a longer-term follow-up study evaluating the longevity and outcomes of the ATTUNE TKA is needed.

## Supplementary Information

**Additional file 1 Supplementary Table 1.** Subgroup analysis of performance-based measures in the PFC Sigma and ATTUNE systems compared between those using and not using a gait aid. **Supplementary Table 2.** Subgroup analysis of performance-based measures in the PFC Sigma and ATTUNE systems compared between CCI score 0–3 and CCI score > 3

**Additional file 2 Supplementary Fig. 1.** The mean values of each performance-based measure in the PFC Sigma and ATTUNE groups compared between those using and not using a gait aid, and within group *p*-values to compare data at baseline, 3 months, 1 year, and 5 years postoperatively. (A) Timed Up and Go test; (B) Two-minute walk test (The data were analyzed using analysis of covariance (ANCOVA).

## Data Availability

All data generated or analysed during this study are included in this published article and its supplementary information files. The data collected in the current study are available from the corresponding author on reasonable request.

## Abbreviations

**PFC**: Press Fit Condylar
**TKA**: total knee arthroplasty
**TUG**: Timed Up and Go
**2MWT**: 2-min walk test
**ROM**: range of motion
**FB**: fixed-bearing
**RP**: rotating-platform
**CR**: cruciate-retaining
**PS**: posterior-stabilized
**OA**: osteoarthritis
**NSAIDs**: non-steroidal anti-inflammatory drugs
**NRS-pain**: numerical rating scale-pain
**BMI**: body mass index
**CCI**: Charlson Comorbidity Index
**HKA**: hip knee axis
**KSS**: Knee Society Score
**WOMAC**: Western Ontario and McMaster University Osteoarthritis Index
**MCID**: minimal clinically important difference
**ANCOVA**: analysis of covariance
**PROMs**: patient-reported outcome measures
