# Supplementary material for: Midterm functional recovery of Total knee arthroplasty patients compared between the ATTUNE knee system and the press fit condylar (PFC) SIGMA knee system
Source: BMC Musculoskelet Disord. 2021 Jul 13;22:620. doi: 10.1186/s12891-021-04464-6 (PMC8278715; doi:10.1186/s12891-021-04464-6)
Supplement: Supplementary file 1 — Additional file 1 Supplementary Table 1. Subgroup analysis of performance-based measures in the PFC Sigma and ATTUNE systems compared between those using and not using a gait aid. Supplementary Table 2. Subgroup analysis of performance-based measures in the PFC Sigma and ATTUNE systems compared between CCI score 0–3 and CCI score > 3 [file 12891_2021_4464_MOESM1_ESM.docx]

| Outcome measurement | With gait aid | | *p*-value | Without gait aid | | *p*-value |
| --- | --- | --- | --- | --- | --- | --- |
|  | PFC Sigma  (n=26) | ATTUNE  (n=34) |  | PFC Sigma  (n=33) | ATTUNE  (n=20) |  |
| **Timed Up and Go test**  Preoperative  3 months  1 year  5 years | 37.7±20.3  26.3±15.5  22.6±15.5  20.8±13.3 | 32.5±19.7  24.7±12.1  19.6±9.6  18.3±6.3 | 0.316  0.668  0.391  0.447 | 15.6±3.7  15.1±6.3  12.6±3.7  14.0±4.5 | 16.6±6.2  15.7±7.1  12.9±1.6  15.9±7.4 | 0.488  0.759  0.747  0.327 |
| **Two-minute walk test**  Preoperative  3 months  1 year  5 years | 27.7±15.8  44.0±20.2  51.2±21.4  51.4±24.7 | 33.8±14.9  45.3±16.7  53.6±16.2  56.9±15.1 | 0.130  0.801  0.653  0.402 | 58.1±18.6  67.8±18.8  76.7±15.5  71.6±18.0 | 57.1±16.2  65.4±14.8  73.4±10.4  68.3±15.5 | 0.842  0.639  0.444  0.548 |

**Supplementary Table 1.** Subgroup analysis of performance-based measures in the PFC Sigma and ATTUNE systems compared between those using and not using a gait aid

Data presented as mean **±** standard deviation

Student’s unpaired *t*-test was used to compare differences in each outcome between the PFC Sigma and ATTUNE systems at each follow-up time point.

A *p*-value<0.05 indicates statistical significance.

**Abbreviation:** PFC, Press Fit Condylar

**Supplementary Table 2.** Subgroup analysis of performance-based measures in the PFC Sigma and ATTUNE systems compared between CCI score 0-3 and CCI score >3

| Outcome measurement | CCI 0-3 | | *p*-value | CCI >3 | | *p*-value |
| --- | --- | --- | --- | --- | --- | --- |
|  | PFC Sigma  (n=35) | ATTUNE  (n=24) |  | PFC Sigma  (n=24) | ATTUNE  (n=30) |  |
| **Timed Up and Go test**  Preoperative  3 months  1 year  5 years | 23.4±13.2  18.5±11.3  16.8±12.6  16.8±11.8 | 21.8±19.3  16.5±7.3  14.4±3.0  14.0±2.3 | 0.621  0.461  0.384  0.334 | 28.3±22.5  21.8±13.9  16.8±9.6  16.9±5.2 | 30.4±21.4  24.9±12.5  19.7±10.6  20.0±8.2 | 0.721  0.404  0.343  0.196 |
| **Two-minute walk test**  Preoperative  3 months  1 year  5 years | 45.7±21.3  59.2±23.9  67.4±22.0  67.1±23.9 | 49.7±19.0  62.3±15.8  67.2±12.9  71.1±10.2 | 0.463  0.583  0.970  0.510 | 43.3±25.8  55.0±20.4  63.9±22.4  57.2±20.8 | 36.6±17.1  45.7±17.7  55.1±18.7  53.9±16.1 | 0.262  0.089  0.155  0.595 |

Data presented as mean **±** standard deviation

Student’s unpaired *t*-test was used to compare differences in each outcome between the PFC Sigma and ATTUNE systems at each follow-up time point.

A *p*-value<0.05 indicates statistical significance.

**Abbreviations:** PFC, Press Fit Condylar; CCI, Charlson Comorbidity Index
