# Supplementary figures and images for: Midterm functional recovery of Total knee arthroplasty patients compared between the ATTUNE knee system and the press fit condylar (PFC) SIGMA knee system
Source: BMC Musculoskelet Disord. 2021 Jul 13;22:620. doi: 10.1186/s12891-021-04464-6 (PMC8278715; doi:10.1186/s12891-021-04464-6)

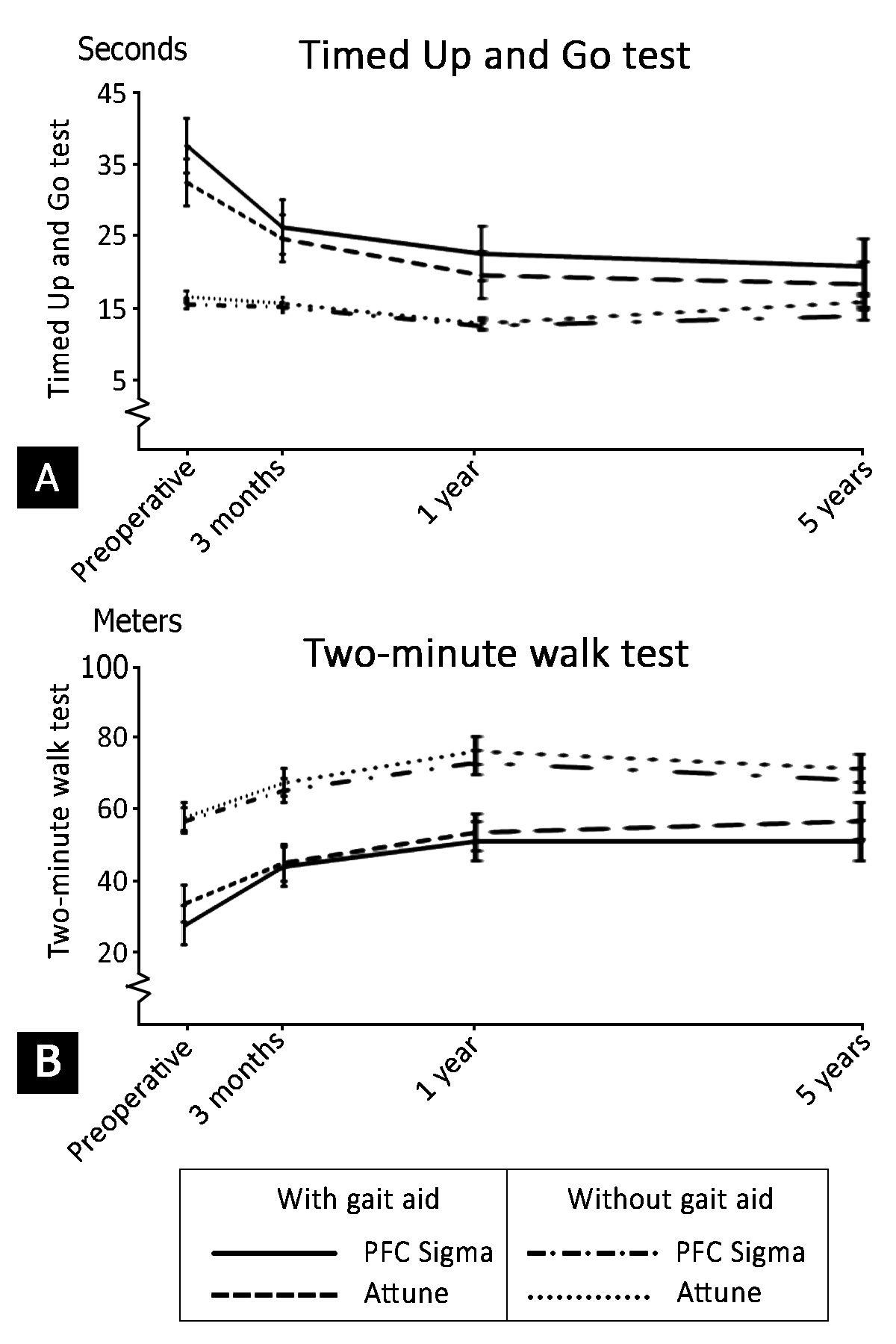

Supplement: Supplementary file 2 — Additional file 2 Supplementary Fig. 1. The mean values of each performance-based measure in the PFC Sigma and ATTUNE groups compared between those using and not using a gait aid, and within group p-values to compare data at baseline, 3 months, 1 year, and 5 years postoperatively. (A) Timed Up and Go test; (B) Two-minute walk test (The data were analyzed using analysis of covariance (ANCOVA). [file 12891_2021_4464_MOESM2_ESM.tif]
